# Host Range Restriction of Insect-Specific Flaviviruses Occurs at Several Levels of the Viral Life Cycle

**DOI:** 10.1128/mSphere.00375-16

**Published:** 2017-01-11

**Authors:** Sandra Junglen, Marvin Korries, Wolfgang Grasse, Janett Wieseler, Anne Kopp, Kyra Hermanns, Moises León-Juárez, Christian Drosten, Beate Mareike Kümmerer

**Affiliations:** a Institute of Virology, University of Bonn Medical Centre, Bonn, Germany; b Robert Koch Institute, Berlin, Germany; c German Centre for Infection Research (DZIF), partner site Bonn–Cologne, Bonn, Germany; The University of Chicago

**Keywords:** insect-specific flavivirus, host range restriction, infection barriers

## Abstract

The genus *Flavivirus* contains emerging arthropod-borne viruses (arboviruses) infecting vertebrates, as well as insect-specific viruses (ISVs) (i.e., viruses whose host range is restricted to insects). ISVs are evolutionary precursors to arboviruses. Knowledge of the nature of the ISV infection block in vertebrates could identify functions necessary for the expansion of the host range toward vertebrates. Mapping of host restrictions by complementation of ISV and arbovirus genome functions could generate knowledge critical to predicting arbovirus emergence. Here we isolated a novel flavivirus, termed Niénokoué virus (NIEV), from mosquitoes sampled in Côte d’Ivoire. NIEV groups with insect-specific flaviviruses (ISFs) in phylogeny and grows in insect cells but not in vertebrate cells. We generated an infectious NIEV cDNA clone and a NIEV reporter replicon to study growth restrictions of NIEV in comparison to yellow fever virus (YFV), for which the same tools are available. Efficient RNA replication of the NIEV reporter replicon was observed in insect cells but not in vertebrate cells. Initial translation of the input replicon RNA in vertebrate cells was functional, but RNA replication did not occur. Chimeric YFV carrying the envelope proteins of NIEV was recovered via electroporation in C6/36 insect cells but did not infect vertebrate cells, indicating a block at the level of entry. Since the YF/NIEV chimera readily produced infectious particles in insect cells but not in vertebrate cells despite efficient RNA replication, restriction is also determined at the level of assembly/release. Taking the results together, the ability of ISF to infect vertebrates is blocked at several levels, including attachment/entry and RNA replication as well as assembly/release.

**IMPORTANCE** Most viruses of the genus *Flavivirus*, e.g., YFV and dengue virus, are mosquito borne and transmitted to vertebrates during blood feeding of mosquitoes. Within the last decade, an increasing number of viruses with a host range exclusively restricted to insects in close relationship to the vertebrate-pathogenic flaviviruses were discovered in mosquitoes. To identify barriers that could block the arboviral vertebrate tropism, we set out to identify the steps at which the ISF replication cycle fails in vertebrates. Our studies revealed blocks at several levels, suggesting that flavivirus host range expansion from insects to vertebrates was a complex process that involved overcoming multiple barriers.

## Abstract (precis)

Most viruses of the genus *Flavivirus*, e.g., YFV and dengue virus, are mosquito borne and transmitted to vertebrates during blood feeding of mosquitoes. Within the last decade, an increasing number of viruses with a host range exclusively restricted to insects in close relationship to the vertebrate-pathogenic flaviviruses were discovered in mosquitoes. To identify barriers that could block the arboviral vertebrate tropism, we set out to identify the steps at which the ISF replication cycle fails in vertebrates. Our studies revealed blocks at several levels, suggesting that flavivirus host range expansion from insects to vertebrates was a complex process that involved overcoming multiple barriers.

## INTRODUCTION

Arthropods can be infected with a wide taxonomic variety of viruses (1). Most of these viruses are transmitted only among arthropods and do not infect other hosts (arthropod-specific viruses). Interestingly, several virus families also contain viruses that are transmitted to vertebrate hosts during the blood-feeding process of the arthropod (arthropod-borne viruses; briefly, arboviruses). Arboviruses have the unique ability to circulate between invertebrate and vertebrate hosts. Infections with arboviruses such as dengue virus (DENV), Chikungunya virus, and Rift Valley fever virus can cause severe disease in humans and animals (2). Coancestral representation of arboviruses and arthropod-specific viruses is known in the RNA virus families *Flaviviridae*, *Togaviridae*, *Bunyaviridae*, *Rhabdoviridae*, *Orthomyxoviridae*, and *Reoviridae* (3, 4).

The evolutionary relationships between arthropod-specific viruses and arboviruses have been unclear. Recent studies on the evolution of bunyavirus host associations suggested that ancestral taxa in this family may have been arthropod specific (5). Host range expansion to vertebrates (arboviral emergence) would have occurred by convergence during the evolution of the four vertebrate-pathogenic genera (genera *Hantavirus*, *Orthobunyavirus*, *Phlebovirus*, and *Nairovirus*). An evolution of arboviruses from arthropod-specific viruses is also assumed for members of other taxa such as the genus *Flavivirus* (family *Flaviviridae*) (6, 7). The vertebrate-infecting flaviviruses, including mosquito-borne flaviviruses (MBF) and tick-borne flaviviruses (TBF) as well as flaviviruses with no known vector (NKV), are all placed in a phylogenetic sister relationship with the classical insect-specific flaviviruses (cISF) (8). However, we and others have recently discovered a novel group of ISFs, named dual-host-affiliated ISFs (dISF), that phylogenetically group within the MBF, making arbo-flaviviruses a paraphyletic group (8–11). Paraphily suggests that vertebrate tropism has been convergently acquired at least two times in flaviviruses.

Flaviviruses are enveloped, positive-stranded RNA viruses. Their genome encodes a single open reading frame (ORF) that is flanked by a 5′ untranslated region (5′ UTR) and a 3′ UTR of about 100 and 350 to 1,200 nucleotides (nt), respectively (12). The ORF encodes one large polyprotein that is co- and posttranslationally cleaved to release the viral proteins, namely, the three structural proteins capsid (C) protein, premembrane/membrane (prM/M) protein, and envelope (E) protein, as well as the nonstructural proteins NS1, NS2A, NS2B, NS3, NS4A, NS4B, and NS5.

In spite of extensive knowledge of flavivirus replication, little is known about the viral proteins and the stages of the replication cycle involved in ISF host range restriction. So far, the host range restriction of ISVs has been studied using Kamiti River virus (KRV), an ISF, and Eilat virus (EILV), an ISV of the genus *Alphavirus* within the family *Togaviridae*. Studies on the ISF Kamiti River virus suggest that the vertebrate innate immune system does not seem to represent a major block, as only a low level of viral RNA and infectious particle shedding was observed in IRF3,5,7^−/−^ mouse embryonic fibroblasts (13). EILV is in a sister relationship with vertebrate-infecting alphaviruses of the Western equine encephalitis virus (WEEV) complex (14). Studies of the replication of an EILV replicon, as well as chimerized constructs in which EILV protein domains were replaced by homologous domains of the arbo-alphavirus Sindbis virus (SINV), revealed that EILV is blocked at two independent stages of the replication cycle, namely, RNA replication and viral entry (14, 15). The latter findings were further supported by *in vivo* infection experiments in mosquitoes and newborn mice. Understanding the restrictions may reveal novel antiviral targets in vertebrates and open novel approaches for virus attenuation and vaccine development, as well as identify determinants of arboviral pathogenicity in humans and livestock.

As insect-specific viruses are described within different virus families, restrictions in host range may occur at different levels of replication cycles depending on the virus taxon. To further understanding of vertebrate tropism restriction in flaviviruses, we provide for the first time results of a replication study on an ISF chimerized with arbo-flavivirus genome elements. These studies were done on a novel flavivirus termed Niénokoué virus (NIEV) that groups with cISF and is a contemporary isolate which has not undergone passage-derived adaptation to cell culture. Chimerization of NIEV with the prototypic arbo-flavivirus yellow fever virus (YFV) is based on a novel NIEV reverse genetics system.

## RESULTS

### NIEV isolation and sequence analysis.

**FIG 1:**
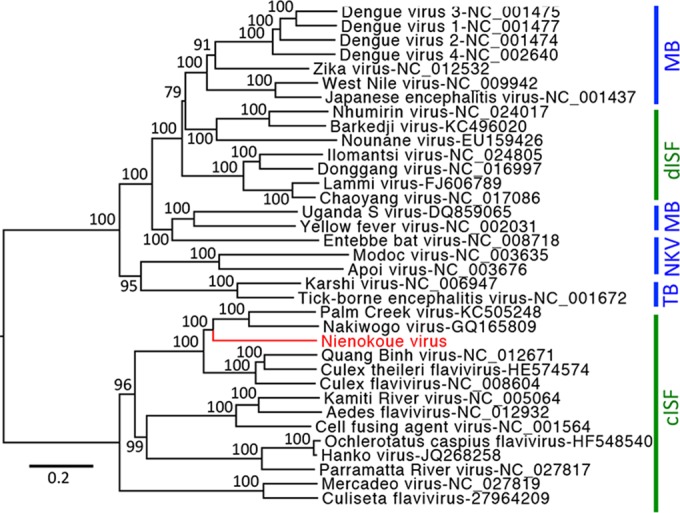
Phylogenetic relationships of NIEV. Complete flavivirus polyproteins were aligned in geneious using MAFFT and the E-INS-I algorithm. Alignment columns were stripped to contain gaps of less than 20%. Maximum likelihood analyses were inferred with the LG substitution matrix and 1,000 bootstrap replicates using PhyML. Nodes are labeled with bootstrap support using percentage values. Vertebrate-pathogenic viruses are labeled with blue vertical lines and insect-specific viruses with green. Abbreviations are as follows: MB, mosquito-borne flaviviruses; TB, tick-borne flaviviruses; NKV, flaviviruses with no known vector; dISF, dual-host-affiliated insect-specific flaviviruses; cISF, classical insect-specific flaviviruses.

**FIG 2:**
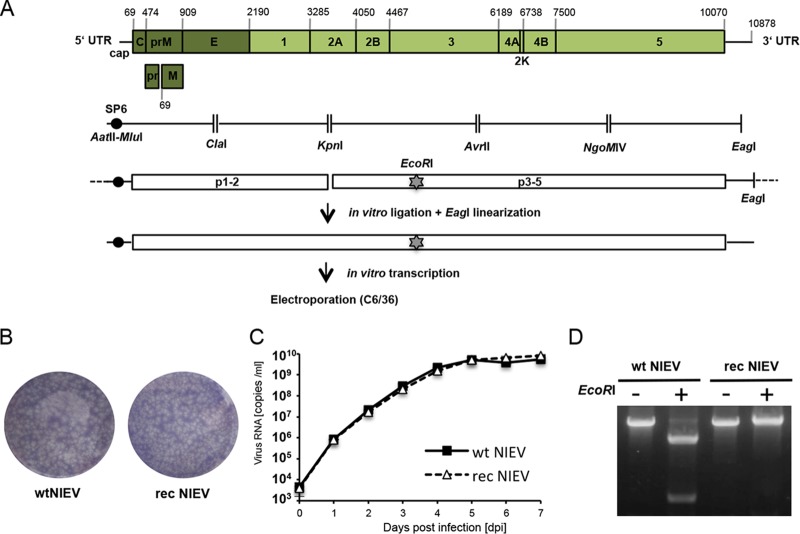
Generation and *in vitro* characterization of recombinant NIEV. (A) Schematic drawing of the NIEV genome organization and reverse genetics system. Depicted nucleotide positions indicate the borders of the encoded viral proteins or the genome end. Below the NIEV genome organization, the reverse-transcribed NIEV cDNA fragments, including the restriction sites used for cloning, are shown. An SP6 promoter sequence was fused to the 5′ end of the NIEV genome. EcoRI indicates the silent genetic marker mutation resulting in deletion of an EcoRI restriction site. After assembly of the cDNA fragments into two plasmids, *in vitro* ligation was performed followed by EagI linearization of the ligation product. The latter was transcribed *in vitro*, and the transcribed RNA was electroporated into C6/36 cells to recover rec NIEV. (B) Plaque morphology of wt NIEV and rec NIEV. Plaque morphology was analyzed by ICA in C6/36 cells using a tragacanth overlay. At 7 days postelectroporation, cells were fixed and subjected to crystal violet staining. (C) Growth kinetics of wt NIEV and rec NIEV. C6/36 cells were infected at an MOI of 0.1. Quantification of viral genome copies in the supernatant was performed by real-time PCR. Data represent means and ranges of results of duplicate infection experiments. (D) Verification of the genetic marker introduced into rec NIEV. Viral RNA was isolated from supernatants of cells infected with the indicated viruses and used as the template for RT-PCR spanning the region containing the deleted EcoRI site in rec NIEV. RT-PCR products were loaded either directly (−) or after EcoRI restriction (+) on an ethidium bromide-stained agarose gel.

Three strains of a novel flavivirus termed NIEV were isolated from *Culex* species mosquitoes collected in the Taï National Park in Côte d’Ivoire. The NIEV genome (strain B51/CI/2004) comprised 10,878 nt. Polyprotein sequence identity with other ISF was 38% to 57%. NIEV shows a genome organization typical for flaviviruses, with three putative structural and seven putative nonstructural proteins (see Fig. 2A). Protein cleavage sites were found to be conserved between NIEV and other flaviviruses. ISFs express an additional protein, named fifo (from fairly interesting flavivirus ORF), by a programed −1 ribosomal frameshift in the NS2A-NS2B gene regions (16). A slippery heptanucleotide was identified for NIEV in this region (G UUU UUU), suggesting a putative fifo protein of 221 amino acids (aa) (8). Phylogenetic analyses based on the complete polyprotein showed that NIEV groups within the primarily *Culex*-associated viruses of the cISF clade (Fig. 1). NIEV defines a new species in basal relationship to Nakiwogo virus from Uganda and to Palm Creek virus from Australia.

In addition to the C6/36 cells used for initial virus isolation, common vertebrate cell lines (Vero, BHK, and HEK-293) that are permissive for all or most arbo-flaviviruses were inoculated with NIEV. No growth was observed in any experiment (data not shown).

### Establishment of a NIEV reverse genetics system.

As a prerequisite to analysis to determine at which level(s) the vertebrate host range restriction of NIEV occurs, we established a NIEV reverse genetics system. Five reverse transcriptase (RT)-PCR fragments covering the complete NIEV genome were amplified from total RNA of infected cells and cloned into individual plasmids. During initial RT-PCR, an SP6 promoter sequence was added upstream of the 5′ end of the viral cDNA and an EagI linearization side was introduced downstream of the 3′ end of the viral cDNA. The individual plasmids were fused stepwise, resulting in two plasmids covering the complete viral sequence. Full-length templates were generated by *in vitro* ligation of the two plasmids (17) (Fig. 2A). RNA transcribed from the EagI-linearized ligation product yielded recombinant (rec) NIEV upon electroporation into C6/36 cells. Recombinant virus produced plaques on C6/36 cells similar to those produced by wild-type (wt) NIEV (Fig. 2B). In growth curve analyses, wt and rec NIEV grew comparably on C6/36 cells (Fig. 2C). The production of rec NIEV was demonstrated by the loss of an EcoRI site through an engineered silent A-to-G mutation at position 5018 (Fig. 2D).

### Host range restriction at the level of RNA replication.

**FIG 3:**
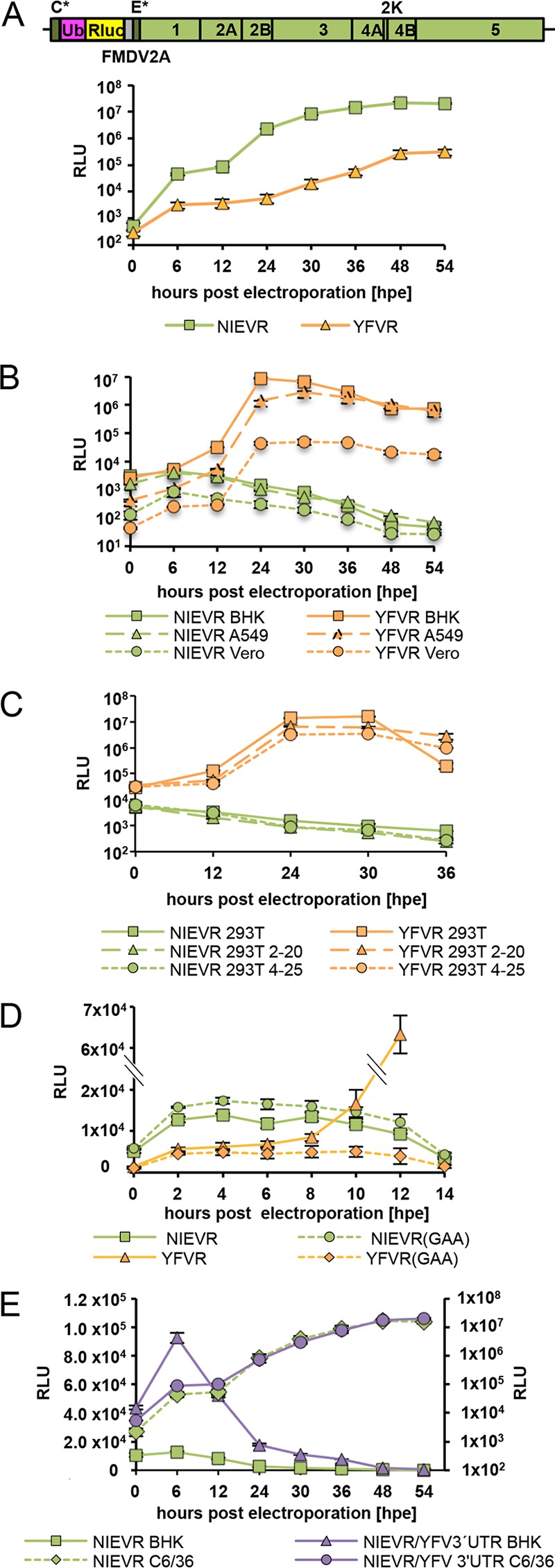
Analyses of NIEV replication and translation. (A) Top: schematic drawing of the NIEV *Renilla* replicon. The narrow dark gray boxes represent the remaining structural proteins, namely, the first 30 amino acids of the capsid protein (C*) and the last 23 amino acids of the E protein (E*), which serve as signal sequence for the following NS1 protein. The pink box indicates the ubiquitin protein (Ub) responsible for generation of the N terminus of the *Renilla* luciferase protein (Rluc; yellow box). The narrow gray box represents the 17-amino-acid-residue autoproteolytic peptide from the foot-and-mouth disease virus (FMDV2A), which cleaves at its own C terminus. Lines indicate the 5′ and 3′ UTRs. Bottom: replication of the NIEV *Renilla* replicon (NIEVR) in insect cells. C6/36 cells were electroporated with NIEVR RNA transcribed *in vitro*. For comparison, electroporation of YFV *Renilla* replicon RNA (YFVR) was performed. Cells were incubated at 28°C, and replication kinetics were monitored by analysis of replicon-derived *Renilla* luciferase expression at the indicated times p.e. Data represent means and ranges of results of duplicate electroporation experiments presented in this panel and in panels B to D. (B) Replication of NIEVR and YFVR in vertebrate cells. The indicated different vertebrate cells were electroporated with *in vitro*-transcribed NIEVR and YFVR RNAs. Cells were incubated at 37°C, and replication kinetics were monitored as described for panel A. (C) Replication of NIEVR and YFVR in Dicer- and miRNA-deficient 293T cells. Wild-type 293T cells or Dicer- and miRNA-deficient cell clones 2–20 or 4–25 were electroporated with *in vitro*-transcribed NIEVR and YFVR RNAs. Cells were incubated at 37°C, and replication kinetics were monitored as described for panel A. (D) Analysis of NIEVR translation. BHK cells were electroporated with replication-incompetent *Renilla* replicon RNAs bearing the exchange of the polymerase GDD motif to GAA, resulting in NIEVR(GAA) or YFVR(GAA). Wild-type NIEVR and YFVR RNAs were electroporated for comparison. Cells were incubated at 37°C, and replication kinetics were monitored as described for panel A. (E) Analysis of NIEVR/YFV 3′ UTR translation. BHK or C6/36 cells were electroporated with NIEVR containing a YFV 3′ UTR insertion downstream of its ORF stop codon. Wild-type NIEVR was electroporated for comparison. Replication kinetics were monitored as described for panel A. Data represent means and ranges of results of duplicate electroporation experiments. Left axis, BHK; right axis, C6/36.

In addition to an infectious full-length cDNA clone, we established a NIEV reporter replicon expressing *Renilla* luciferase (Fig. 3A). The latter allows circumventing the entry step, thereby enabling separate analyses of viral RNA replication and translation. The functionality of the NIEV replicon was confirmed by *Renilla* luciferase expression upon electroporation in insect cells, generating levels of luminescence that exceeded those of an established YFV replicon (Fig. 3A) (18). While the analogous YFV replicon efficiently replicated in different vertebrate cells (BHK, A549, Vero), only a slight increase in *Renilla* luciferase levels was observed at 6 h postelectroporation (p.e.) for the NIEV replicon (Fig. 3B) before a continuous decrease was observed. NIEV RNA replication could not be rescued in Dicer- and microRNA (miRNA)-deficient 293T cells (Fig. 3C) (19), while YFV RNA replication was still supported by these cells. These results indicate a restriction of NIEV in vertebrate cells at the level of RNA replication and suggest that vertebrate cell-specific miRNAs are unlikely to constitute a sufficient factor for the observed NIEV host range restriction.

To further evaluate whether the slight increase of luciferase levels observed at 6 h p.e. is due to a low level of RNA replication or to initial translation, we created a replication-incompetent version of the NIEV replicon in which the catalytic GDD motif of the RNA-dependent RNA polymerase had been mutated to GAA. *Renilla* luciferase expression levels were measured in short intervals immediately after electroporation. Similar extents of signal increase were observed for the two versions of the replicon, indicating that luciferase expression was caused by direct translation of incoming RNA rather than by RNA replication (Fig. 3D). For comparison, while a YFV replicon GAA variant also showed a slight initial increase in *Renilla* luciferase signal, the luciferase signal from the replicating wt YFV replicon increased exponentially from 8 h p.e. (Fig. 3D). It was concluded that NIEV replication was blocked in vertebrate cells at a stage downstream of initial genome translation.

Since it was previously reported that insertion of a SINV 3′ UTR motif into the 3′ UTR of sleeping disease virus (SDV) increased SDV translation efficiency in a cell-specific manner (20), we performed similar analyses by inserting the YFV 3′ UTR downstream of the NIEV ORF stop codon and analyzing the result for enhanced translation in BHK cells. Although the chimeric NIEV *Renilla* replicon (NIEVR)/YFV 3′ UTR was still unable to replicate in BHK cells, its translation efficiency increased about 2-fold faster in BHK cells within the first 6 h p.e. than that of the wt NIEV replicon (Fig. 3E). This suggests that the arbo-flavivirus 3′ UTR might be involved in vertebrate tropism.

### Host range restriction at the level of cell entry.

**FIG 4:**
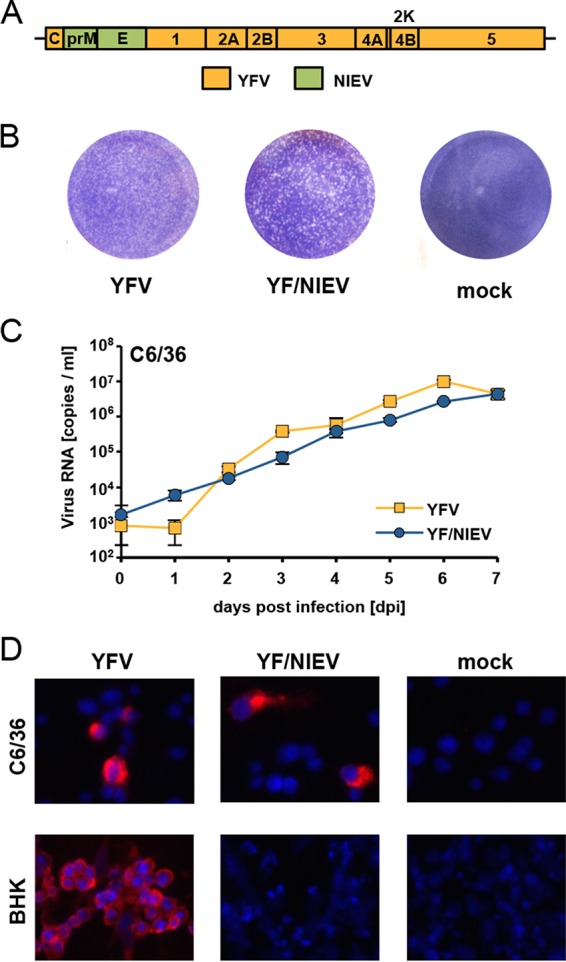
Construction and characterization of YF/NIEV chimera. (A) Schematic drawing of the YF/NIEV chimera. The envelope proteins prM and E of YFV were exchanged against the corresponding prM and E proteins of NIEV (green boxes). (B) Plaque morphology of YFV and YF/NIEV. C6/36 cells were electroporated with *in vitro* RNA transcripts of the indicated constructs or were mock transfected and processed for infectious center assay analyses. Seeded cells were overlaid with tragacanth. At 7 days p.e., cells were fixed and subjected to crystal violet staining. (C) Growth kinetics of YFV and YF/NIEV. C6/36 cells were infected at an MOI of 0.1. Quantification of viral genome copies in the supernatant was performed by real-time PCR. Data represent means and ranges of results of duplicate infection experiments. (D) Immunofluorescence analyses after infection of different cells. C6/36 or BHK cells were infected with the indicated viruses at an MOI of 0.1. At 48 h p.i., immunofluorescence analysis was performed using a monoclonal anti-YFV NS1 antibody. Nuclei were DAPI stained.

**FIG 5:**
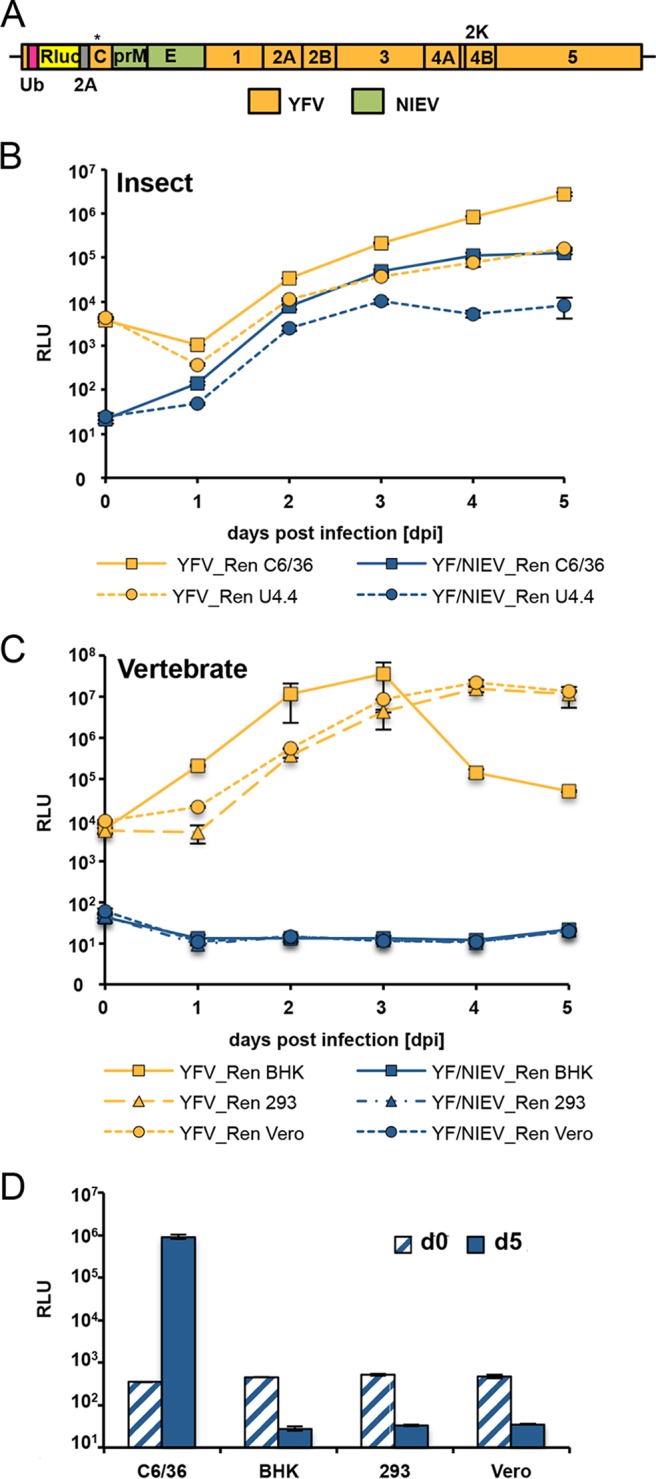
Establishment and growth characteristics of an infectious YF/NIEV reporter chimera. (A) Schematic drawing of the YF/NIEV chimera expressing *Renilla* luciferase. An Rluc gene flanked by sequences encoding ubiquitin and FMDV2A was inserted after the first 30 codons of the capsid gene. The full-length capsid gene encoding a silently mutated cyclization sequence (denoted by an asterisk [*]) to limit long-range interactions with the 3′ cyclization sequence was fused downstream of the cassette. (B) Growth kinetics of YFV *Renilla* reporter virus (YFV_Ren) and YF/NIEV *Renilla* reporter virus (YF/NIEV_Ren) in insect cells. C6/36 or U4.4 cells were infected with the reporter viruses at an MOI of 1, and *Renilla* luciferase levels were determined at the indicated time points p.i. Data represent means and ranges of results of duplicate infection experiments presented in this panel and in panels C and D. (C) Growth kinetics of YFV_Ren and YF/NIEV_Ren in vertebrate cells. BHK, 293T, and Vero cells were infected with the reporter viruses at an MOI of 1 followed by *Renilla* luciferase readout at the indicated time points p.i. (D) Growth analyses of YF/NIEV_Ren in different cells. C6/36, BHK, 293T, and Vero cells were infected at an MOI of 10. *Renilla* luciferase levels were determined at 5 days p.i.

To analyze whether the NIEV host range restriction is also manifested at the level of cell entry, we replaced the envelope protein prM and E genes with the homologous genes of NIEV in a YFV infectious cDNA clone, resulting in a chimeric construct termed YF/NIEV (Fig. 4A). Our construct contains the YFV capsid protein along with the YFV nonstructural proteins to ensure cleavage at the C protein anchor site by the NS2B-3 protease, which is a prerequisite for signalase-mediated cleavage at the C-prM junction (21). Electroporation of the *in vitro*-transcribed YF/NIEV RNA into C6/36 cells (22) resulted in formation of plaques in an infectious center assay (ICA), indicating the production of infectious particles (Fig. 4B). Infectious particle production was further proven in growth curve analyses after infection of C6/36 cells (Fig. 4C). Immunofluorescence analysis using a YFV anti-NS1 antibody showed that C6/36 cells were infected with both YFV and YF/NIEV (Fig. 4D). In contrast, no positive immunofluorescence signal was observed after infection of BHK cells with YF/NIEV (Fig. 4D), indicating that the structural proteins of NIEV are unable to mediate infection of BHK cells. To comparatively test further vertebrate cells for infection with YFV and YF/NIEV, we established YFV and YF/NIEV *Renilla* reporter viruses (YFV_Ren and YF/NIEV_Ren). To this end, a *Renilla* gene cassette was inserted in frame in the N-terminal region of the YFV or YF/NIEV polyprotein in a manner similar to that previously described for DENV (23) (Fig. 5A). After infection of insect cells (C6/36 and U4.4) with the reporter viruses YFV_Ren and YF/NIEV_Ren at a multiplicity of infection (MOI) of 1, luciferase levels increased over time as expected for viable reporter viruses (Fig. 5B). In several different vertebrate cell cultures, including cultures of BHK, HEK-293, and Vero cells, YFV_Ren but not YF/NIEV_Ren caused luciferase expression (MOI of 1) (Fig. 5C). Infection of vertebrate cells with YF/NIEV_Ren was also not observed at an increased MOI of 10 (Fig. 5D). The structural proteins of NIEV appeared to be unable to mediate entry into vertebrate cells.

### Host range restriction at the level of assembly and release.

**FIG 6:**
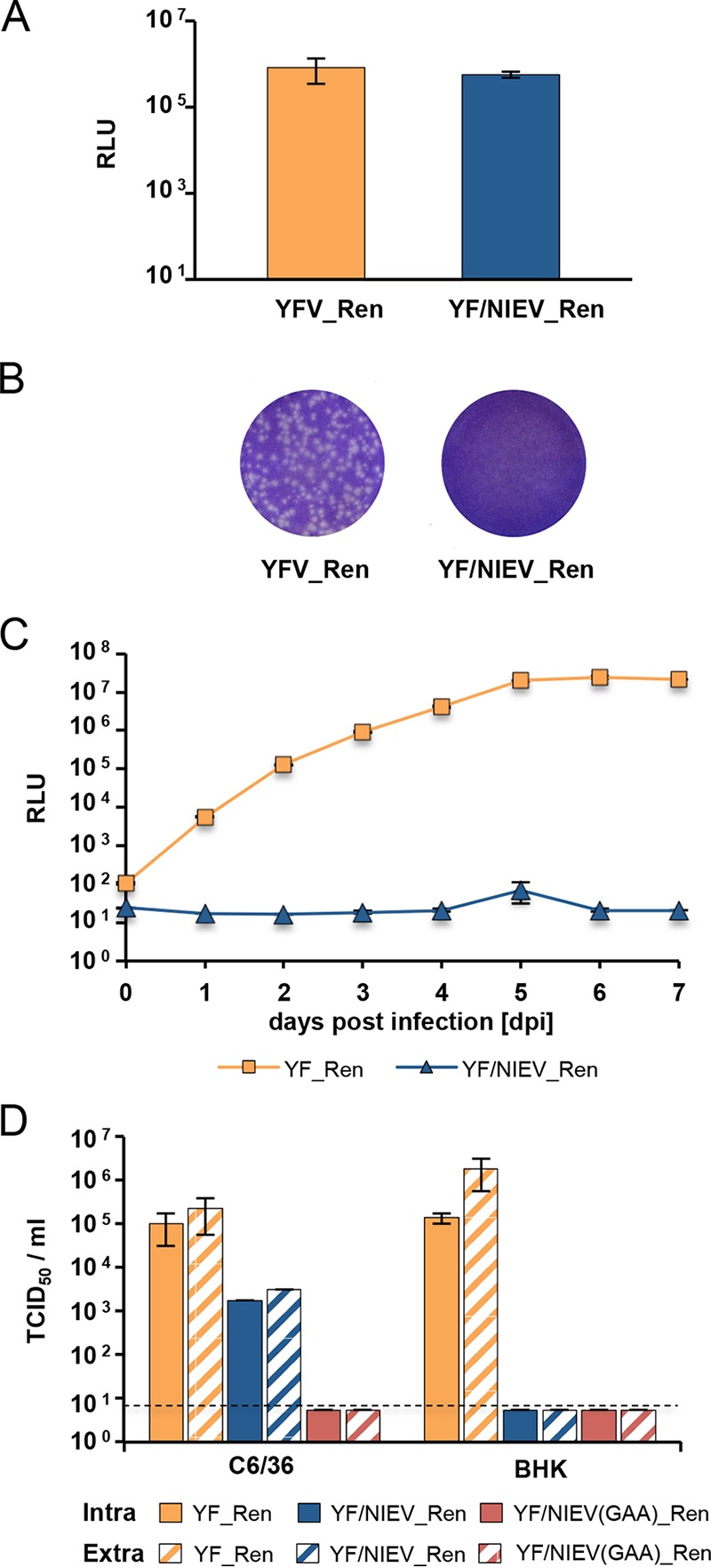
Analyses of the YF/NIEV reporter virus construct in vertebrate cells. (A) RNA replication of YF/NIEV_Ren in BHK cells. BHK cells were electroporated with the indicated *in vitro*-transcribed reporter virus RNAs, and *Renilla* luciferase expression levels were measured at 24 h p.e. Data represent means and ranges of results of duplicate electroporation experiments. (B) Plaque formation of YFV_Ren and YF/NIEV_Ren in BHK cells. BHK cells were electroporated with *in vitro* RNA transcripts of the indicated constructs and processed for ICA analyses. Seeded cells were overlaid with agarose. At 3 days p.e., cells were fixed and subjected to crystal violet staining. (C) Infectivity assay. Supernatants from BHK cells electroporated with either YF_Ren or YF/NIEV_Ren *in vitro*-transcribed RNAs were used to infect C6/36 cells. To test for infectivity, *Renilla* luciferase levels were determined at the indicated time points p.i. Data represent means and ranges of results of duplicate infection experiments. (D) Intra- and extracellular infectivity titers. *In vitro* transcripts from the full-length clones specified at the bottom were electroporated in C6/36 or BHK cells as indicated. At 6 (C6/36) or 2 (BHK) days p.e., cell lysates and supernatants were subjected to three freeze and thaw cycles. Titers were determined by TCID_50_ assay using C6/36 cells and *Renilla* luciferase as the readout. Dashed line, detection limit. Data represent means and ranges of results of duplicate electroporation experiments.

Next, we analyzed whether the NIEV envelope proteins are able to mediate assembly and release of infectious virus in vertebrate cells. To circumvent the level of cell entry, RNA transcribed from reporter virus cDNA clones was directly electroporated into target cells. In BHK cells, YF/NIEV_Ren RNA yielded luciferase expression levels similar to those seen with YFV_Ren, indicating efficient RNA replication and protein expression (Fig. 6A). However, no plaques were observed for YF/NIEV_Ren in an infectious center assay (Fig. 6B), suggesting a lack of infectious particle production. Absence of released infectious viral progeny was confirmed by passaging of supernatants, obtained upon electroporation of BHK cells, in C6/36 cells. While an increase of luciferase values over time demonstrated passage of infectious particles for YFV_Ren, no luciferase expression was seen for YF/NIEV_Ren (Fig. 6C).

To further investigate whether infectious virus exists in intracellular locations, C6/36 and BHK cells were electroporated with YF_Ren or YF/NIEV_Ren *in vitro* transcripts and titers of infectious virus contained in cells were measured in addition to levels of released particles by endpoint dilution assay on C6/36 cells. YF/NIEV_Ren(GAA) bearing the exchange of the polymerase GDD motif to GAA was included as a negative control. Due to different replication rates of YFV in C6/36 and BHK cells and the occurrence of a strong cytopathic effect in BHK cells at day 3 (d3) postinfection (p.i.), titers of infectious virus were determined at different time points. As shown in Fig. 6D, intracellular particles were detected for YF_Ren in BHK and C6/36 cells at d2 and d6, respectively. While YF/NIEV_Ren also produced infectious intracellular particles in C6/36 cells, no intracellular infectivity was measured in BHK cells. Prolonged incubation of BHK cells until d6 p.e. also did not result in the recovery of infectious intracellular particles (data not shown). In accordance with the results presented in Fig. 6C, infectious extracellular particles were released in all cases where infectious intracellular particles were observed. Hence, NIEV envelope proteins appeared to be unable to mediate assembly of infectious intracellular particles and, consequently, no infectious particles were released in vertebrate cells.

## DISCUSSION

While arboviruses show dual-host tropism with adaptation to insect and vertebrate hosts, insect-specific viruses, including ISF, have a single-host tropism. Here we have discovered NIEV, a new ISF, in mosquitoes from West Africa. NIEV provides a favorable model to study vertebrate host restriction due to its known and limited history of cell culture passages after primary isolation. Vertebrate host restriction may occur on the levels of viral attachment and entry, genome replication, and virus assembly and release. Experiments in the present study resembled earlier work on the replacement of surface proteins in two different arbo-flaviviruses, YFV and DENV, with the surface proteins of the NKV flavivirus Modoc virus (MODV) (24). These virus chimeras replicated efficiently in vertebrate and insect cells, whereas wt MODV does not replicate in insect cells, suggesting that host tropism is not determined by surface proteins (24). Similarly, it was demonstrated that a chimeric DENV genome carrying the envelope genes from Langat virus (LGTV), a tick-borne virus unable to infect mosquito cells, retained the ability to infect mosquito cells irrespective of the type of envelope proteins (25). Hence, the restriction to replicate in mosquito cells is at a postentry event for both MODV and LGTV, which is somewhat surprising considering the known mechanism of receptor-dependent entry for many flaviviruses.

These experiments were directed at permissiveness in insect cells. However, how ISFs are hindered from infecting vertebrate cells is of special interest in studying virus emergence. The only reverse genetics-based data available in this field have been generated recently from EILV, a novel alphavirus related to the WEEV serocomplex of arbo-alphaviruses, where restriction occurs at the levels of both entry and replication (14, 15).

In the present work, reverse genetics of an ISF, namely, NIEV, was used for the first time to study vertebrate restriction, revealing a clear block of entry and RNA replication and assembly in vertebrate cells. For other flaviviruses, several host factors have been described to be involved in RNA replication, either via binding to the viral RNA genome or via binding to viral proteins involved in the replication complex. Among those are proteins such as AUF1 p45 that bind to the viral RNA functioning as RNA chaperons (26). RNA chaperon activity was also assumed for the La autoantigen, whereas a function as an RNA helicase was suggested for the viral RNA binding translation elongation factor-1α (EF-1α) (27, 28). For cyclophilin A, an interaction with both viral RNA and the viral NS5 protein in the replication complex was previously described (29). Similarly, TIA/TIAR colocalize with viral replication complex components, namely, double-stranded RNA (dsRNA) and the NS3 protein (30). A lack of or a difference between such host factors (or their genetic homologs) in the vertebrate cell might account for the observed lack of NIEV RNA replication. Interestingly, for the tomato spotted wilt virus (TSWV), a plant- and insect-infecting *Tospovirus* belonging to the family *Bunyaviridae*, a viral polymerase-bound host factor was identified that rendered human cells permissive with respect to this virus (31).

In addition to proteins, host small noncoding RNAs can influence viral RNA replication (32). Recently, further miRNA interaction studies using cross-linking immunoprecipitation (CLIP) based on *Argonaute* protein have been performed for 15 different viruses (33). Although that study also included arboviruses, functional miRNA interaction studies have been performed so far only in vertebrate cells. Analogous studies for arboviruses and also ISFs in insect cells are awaited. Also, it was described that cellular miRNAs can target viral RNAs during infections, resulting in inhibition of virus replication (34–36). In the context of the present study, we found that vertebrate cell-specific miRNAs are unlikely to represent a factor sufficient for the observed NIEV host range restriction, since NIEV RNA replication did not occur in Dicer- and miRNA-deficient 293T cells also (19), while YFV RNA replication was still supported by these cells.

While RNA replication in vertebrate cells was inhibited for NIEV, translation of incoming RNA still occurred. Recently, it was demonstrated for SINV that its 3′ UTR contains an RNA motif that increases translation of SINV genomic and subgenomic mRNAs in insect cells but not in mammalian cells (20). This SINV RNA motif was also able to increase the replication efficiency of a replicon derived from sleeping disease virus (SDV), an alphavirus without known arthropod vector, in insect cells upon insertion into the SDV replicon 3′ UTR. These findings imply a possible role of the 3′ UTR motif in the virus host range. Similarly, when we inserted the 3′ UTR of YFV directly downstream of the ORF stop codon into the NIEV replicon, its translation efficiency in BHK cells was increased early after electroporation compared to that of the wt NIEV replicon, also suggesting a possible role of the YFV 3′ UTR in host range specificity. This would be in accordance with the findings for DENV, which indicated that certain RNA structure elements in the flavivirus 3′ UTR, like RNA structure duplications, play a role in host adaptation (37, 38). Furthermore, the flavivirus 3′ UTR is the origin of subgenomic flavivirus RNAs (sfRNAs) (39–41). It has been reported that sfRNAs exhibit interferon-antagonistic activities (41, 42). In comparison to, e.g., wt West Nile virus, growth of a West Nile virus mutant deficient in producing sfRNA was reduced in wt mouse embryonic fibroblasts (MEFs), while the mutant replicated like the wt virus in IRF3^−/−^ × IRF7^−/−^ MEFs as well as in Vero and BHK cells, which are also described to be interferon deficient (39, 42, 43). As NIEV did not grow in Vero or BHK cells, the interferon-antagonistic function of an sfRNA does not seem to be a crucial factor with regard to host range restriction. However, sfRNAs have also been described to inhibit the cellular 5′-to-3′ exonuclease XRN1, resulting in increased stability of many cellular mRNAs, or to counteract RNA interference mediated by small interfering RNAs (siRNAs) or miRNAs (44–46). It therefore will be interesting to analyze whether modulation of the host cell/host antiviral response via those mechanisms contributes to host range restriction.

Nevertheless, our data determined using the YF/NIEV chimera expressing the NIEV envelope proteins demonstrate that the first barrier against infection of vertebrate cells by ISF is viral entry. While the YF/NIEV chimera readily grew in insect cells, the recovered virus was unable to infect BHK cells. Also, additional vertebrate cells proved to be resistant to infection, as was shown using a reporter YF/NIEV chimera expressing *Renilla* luciferase. Several studies have been performed to elucidate factors and receptors involved in flavivirus attachment and entry. Still, detailed knowledge of interaction of the flaviviral envelope proteins with bona fide receptors is limited. Among the factors involved in attachment and entry are glycosaminoglycans, different C-type lectins (DC-SIGN/L-SIGN, mannose receptor, CLEC5A, and mosGCTL-1), phosphatidylserine receptors (TIM and TAM), and vitronectin receptor integrin α_ν_β_3_ (47). The majority of the factors have been determined in mammalian cells. Prohibitin and mosGCTL-1 have been described solely in the context of mosquito cells (47). Looking at the available data, it is obvious that individual flaviviruses use several different attachment and entry factors. On the other hand, differential expression of entry factors is already observed among different human cells, indicating the complex interplay between virus and cells involved in tropism. Hence, in-depth studies will be necessary to elucidate what restricts attachment and/or entry of ISFs to vertebrate cells.

In comparison to host factors involved in flavivirus entry, even less is known about host factors important for flavivirus assembly/release. Also, for the other insect-specific virus analyzed with regard to host range restriction, namely, the alphavirus EILV, no data are described analyzing a possible block in assembly and/or release. The respective data would have allowed judging whether an assembly/release block is independent of the budding site, since flaviviruses bud into the endoplasmic reticulum whereas alphaviruses bud from the cell membrane.

Taking the results together, our analyses show that the NIEV vertebrate host range restriction occurs at several levels of the viral life cycle. This suggests that flavivirus host range expansion from insects to vertebrates was a complex process that involved overcoming multiple barriers. The apparent complexity of adaptive barriers makes it unlikely that sudden host range expansions, such as by acquisition of single-site mutations, will occur in ISF. While NIEV belongs to the so-called cISF, which are phylogenetically distinct from all other flaviviruses, dISF have also been described (8). Since the latter phylogenetically group close to the mosquito-borne flaviviruses, it will be of interest to analyze whether those viruses are also restricted at the same levels or whether barriers restricting dISF infection of vertebrate cells might be determined by fewer adaptive steps.

## MATERIALS AND METHODS

### Virus isolation and genome sequencing and analyses.

Mosquito sampling and virus isolation was performed as described before (9, 48).

Three cultures of C6/36 cells that had been inoculated with pools of *Culex* species mosquitoes collected in the Taï National Park in Côte d’Ivoire induced mild cytopathic effects (CPE) 5 days postinfection (p.i.). A fragment of the NS5 gene was amplified and sequenced from each isolate. The obtained sequences showed 99% identity among themselves and less than 60% identity to cISF, suggesting the identification of three strains (strains NIEV-B21/CI/2004, NIEV-B51/CI/2004, and NIEV-C64/CI/2004) of a novel flavivirus, termed NIEV. The complete genome of strain NIEV-B51/CI/2004 and the complete ORF of NIEV-B21/CI/2004 (10,171 nt) as well as a subcomplete ORF of NIEV-C64/CI/2004 (9,864 nt) were sequenced using primer-walking techniques based on genome-specific and generic pan-flavivirus primers. Genome termini were sequenced by rapid amplification of cDNA ends (RACE)-PCR according to the instructions of the manufacturer (Invitrogen, Germany). As the three strains were more than 98% identical, further work was focused on strain NIEV-B51/CI/2004. For phylogenetic analyses, polyprotein ORFs were aligned in geneious using MAFFT and the E-INS-I algorithm. Alignment columns were stripped to contain gaps of less than 20%. Maximum likelihood analyses were inferred with the LG substitution matrix and 1,000 bootstrap replicates using PhyML.

### Generation of recombinant NIEV and NIEV reporter replicon.

C6/36 cells were infected with NIEV, and total cellular RNA was isolated using peqGOLD TriFast (Peqlab). cDNA synthesis was performed with genome-specific primers using SuperScript III reverse transcriptase (Invitrogen). The complete genome of NIEV was amplified in five PCR fragments using Phusion High-Fidelity polymerase and cloned into individual plasmids (Fig. 2A). At the 5′ end, an SP6 promoter sequence was fused together with an upstream AatII and MluI restriction site for cloning. At the 3′ end, an EagI site for linearization was added. The five amplified fragments were individually subcloned using standard procedures. Fragments 1 and 2 were further assembled into the pACYC177 background, resulting in pNIEV1-2, whereas fragments 3 to 5 were assembled into pSmart-LC-Kan, resulting in pNIEV3-5. As we failed to stably fuse all five individual plasmids into the two low-copy-number vectors pSMART and pACYC177, we generated a full-length template by *in vitro* ligation (Fig. 2A). To this end, the MluI-KpnI fragment excised from pNIEV1-2 was ligated into pNIEV3-5 cut with KpnI and MluI.

To generate a NIEV reporter replicon, a cassette encoding the sequences for ubiquitin–*Renilla* luciferase–foot-and-mouth disease virus 2A was amplified from the previously published YFV *Renilla* replicon (18). The resulting fragment was N-terminally fused to the first 30 codons of the NIEV capsid protein, including the SP6 promoter preceded by an MluI restriction site sequence, and C-terminally fused to a fragment encoding the last 23 codons of the E protein up to the genomic KpnI restriction site. To obtain the NIEV reporter replicon, the fusion product was cut with MluI and KpnI and inserted into a Mlu-KpnI-cut pACYC177 vector encoding NIEV fragments 3 to 5. Insertion of the YFV 3′ UTR downstream of the stop codon of the NIEV ORF was done using Fusion PCR technology. Plasmids were verified by sequencing. Primer sequences and further details on cloning can be obtained upon request.

### Generation of reporter and chimeric viruses.

To establish a YFV *Renilla* reporter virus (YF_Ren), two PCR fragments were amplified from pACNR/FLYF-17D using Bo617 (5′ CCAATCCCGGGCCCATGTCTGGTCGTAAAGCTC 3′) and Bo620 (5′ GAACTCCTCGTCGTACCATGTTAACGCCCAGGGTTTTTC 3′) or from Bo619 (5′ GAAAAACCCTGGGCGTTAACATGGTACGACGAGGAGTTC 3′) and Bo618 (5′ GATAGATCCATCGCAGTCTATGGTGTA 3′). The two PCR fragments were fused using Bo617 and Bo618 as amplifying primers. The resulting PCR fragment encoding the FMDV2A cleavage site followed by a full-length capsid gene encoding a silently mutated cyclization sequence to limit long-range interactions with the 3′ cyclization and the 5′ terminal region of the NS1 gene was cut with XmaI and MluI and inserted into the YFV *Renilla* replicon plasmid (18) cut with XmaI and MluI.

For generation of the YF/NIEV chimera, the NIEV prM-E genes were amplified from pNIEV1-2 using primers Bo320 (5′ GGGAATGCTGTTGATGACGGGTGGAGTTGTGGACGTGAACATGAGTTTGG 3′) and Bo323 (5′ CAAAGTTGATGGCGCATCCTTGATCTGCTCGTACATAGTAAATCATGCCC 3′). The resulting fragment was fused N-terminally with a pACNR/FLYF-17D-derived fragment amplified with Bo286 (5′ ACGAGAGAGATGATAGGGTCTGC 3′) and Bo321 (5′ CCAAACTCATGTTCACGTCCACAACTCCACCCGTCATCAACAGCATTCCC 3′) and C-terminally with a pACNR/FLYF-17D-derived fragment amplified with Bo322 (5′ GGGCATGATTTACTATGTACGAGCAGATCAAGGATGCGCCATCAACTTTG 3′) and Bo88 (5′ TTGGAGAGCCATGGGCACTC 3′) via fusion PCR technology using primers Bo286 and Bo88 as amplifying end primers. The obtained fragment was cut with NotI and MluI and inserted into pACNR/FLYF-17D cut with the same enzymes.

The YF/NIEV_Ren reporter virus was established according to the method used with the YF_Ren reporter virus, except that the template for amplification via Bo619 and Bo618 was pYF/NIEV. The integrity of the plasmids was verified by sequencing.

### *In vitro* transcription and electroporation.

The NIEV full-length template obtained via *in vitro* ligation of pNIEV1-2 and pNIEV3-5 and the NIEV reporter replicon plasmid (2 µg) amplified in MC1061 bacteria were linearized with EagI. Plasmids pACNR/FLYF and derivatives were linearized with XhoI. *In vitro* transcriptions were performed using an SP6 mMessage mMachine kit (Applied Biosystems). Electroporation of BHK-21J, A549, and Vero cells was performed as described previously for BHK cells except that the preset HeLa settings were used for the Vero cells (18). C6/36 and U4.4 cells were electroporated as previously described (22).

### Infectious center assays.

ICAs in BHK cells were performed as previously described (49). For ICAs in C6/36 cells, 10-fold serial dilutions of transfected cells were mixed with 2 × 10^6^ untransfected C6/36 cells and seeded in 35-mm-diameter dishes. Following attachment for 4 h, the medium was replaced by Leibovitz 15 media containing 2.5% fetal calf serum (FCS) and 1% tragacanth. Plates were incubated for 7 days and fixed and stained as previously described (49).

### Virus stocks, infectivity assays, and growth kinetics.

YFV17D was derived from infectious full-length clone pACNR/FLYF-17D (kindly provided by Charles M. Rice, NY) by electroporation of *in vitro*-transcribed RNA into BHK cells (50). The NIEV wild-type strain and the YF/NIEV chimeras were cultured in C6/36 cells.

Plaque titration on BHK cells was performed as previously described (49). Plaque assays on C6/36 cells were performed accordingly using 2 × 10^6^ cells seeded the day before in 35-mm-diameter dishes and proceeded using a 1% tragacanth overlay as described for ICAs. For the *Renilla* reporter viruses, viral titers were determined by 50% tissue culture infective dose (TCID_50_) titration using C6/36 cells seeded the day before with 4 × 10^4^ cells/well. Virus-positive wells were identified by *Renilla* luciferase assay at d7 p.i.

To determine intracellular infectivity titers, electroporated cells were disrupted after extensive washing with phosphate-buffered saline (PBS) using three freeze-thaw cycles as previously described (51), except that BHK cell pellets were resuspended in minimum essential medium (MEM) (containing 7.5% FCS and nonessential amino acids [NEAA]) and C6/36 cell pellets in Leibovitz 15 media (containing 5% FCS). For the intracellular infectivity experiment, virus-containing culture supernatants from electroporated cells were subjected to the same three cycles of freezing and thawing and infectivity was determined in parallel by endpoint dilution assay as described above for *Renilla* reporter viruses.

For growth kinetic experiments, C6/36, U4.4., BHK-21J, Vero CCL81, and HEK-293 cells were infected at the indicated multiplicity of infection (MOI) for 1 h at 37°C or 28°C, depending on the cell type/temperature experiment performed. At defined time points after infection, either cell culture supernatants or lysed cells were collected and analyzed via real-time PCR (NIEV) or *Renilla* assay (reporter viruses), respectively.

### RNA preparation and real-time PCRs.

Viral RNA from cell culture supernatants was prepared using a NucleoSpin RNA virus kit (Macherey-Nagel). For NIEV RNAs, cDNA was synthesized using a SuperScript III RT system (Life Technologies, Inc.) and random hexamer primers. Viral genome copy numbers were quantified by real-time RT-PCR using primers B51_TM_F (5′ CATGTGGAGTGGGCGGAATA 3′) and B51_TM_R (5′ TGGGCCAGCTCTAACAGGAA 3′) and probe B51_TM (5′ 6-carboxyfluorescein [FAM]-CCAACCAGTGTTCTTTCCTAGCGATTTCTTC-6-carboxytetramethylrhodamine [TAMRA] 3′).

Quantification of YFV RNA was performed using a One Step RT-PCR protocol essentially as previously described (52).

### *Renilla* assay.

*Renilla* luciferase was measured from lysed infected cells using the *Renilla* luciferase assay system (Promega). Luciferase activity was measured in relative light units (RLU). Measurements were performed using a Junior LB 9509 luminometer (Berthold Technologies).

### Immunofluorescence.

Indirect immunofluorescence analyses were performed using monoclonal antibody 1A5 directed against the YFV NS1 protein (kindly provided by J. J. Schlesinger) and Alexa Fluor 555 goat anti-mouse serum (Thermo Fisher Scientific). Nuclei were stained using 4′,6-diamidin-2-phenylindol (DAPI).

### Accession number(s).

Sequences were deposited at GenBank with accession numbers KX879625, KX879626, and JQ957875.
